# EOS, an Ikaros family zinc finger transcription factor, interacts with the HTLV-1 oncoprotein Tax and is downregulated in peripheral blood mononuclear cells of HTLV-1-infected individuals, irrespective of clinical statuses

**DOI:** 10.1186/s12985-019-1270-1

**Published:** 2019-12-19

**Authors:** Tadasuke Naito, Hiroshi Ushirogawa, Takuya Fukushima, Yuetsu Tanaka, Mineki Saito

**Affiliations:** 1 Department of Microbiology, Kawasaki Medical School, 577 Matsushima, Okayama, 701-0192 Japan; 2 Laboratory of Hematoimmnology, School of Health Sciences, Faculty of Medicine, University of the Ryukyus, 207 Uehara, Okinawa, 903-0215 Japan; 3 Department of Immunology, Graduate School of Medicine, University of the Ryukyus, 207 Uehara, Okinawa, 903-0215 Japan

**Keywords:** HTLV-1, HAM/TSP, EOS (Ikzf4), Tax, HBZ

## Abstract

**Background:**

EOS plays an important role in maintaining the suppressive function of regulatory T cells (Tregs), and induces a regulated transformation of Tregs into T helper-like cells, which are capable of secreting proinflammatory cytokines in response to specific inflammatory signals. Meanwhile, significant reduction in Treg activity along with production of proinflammatory cytokines has been reported in patients with HTLV-1-associated myelopathy/tropical spastic paraparesis (HAM/TSP).

**Methods:**

In this study, to examine whether there is an alteration in EOS expression in peripheral blood mononuclear cells (PBMCs) derived from HTLV-1-infected individuals especially HAM/TSP, we investigated the expression of HTLV-1 tax genotype, proviral load (PVL), and the mRNA expression of tax, HBZ and EOS in HTLV-1 infected individuals including adult T-cell leukemia/lymphoma (ATL), HAM/TSP, or asymptomatic carriers. The expression levels of EOS mRNA and protein in various HTLV-1-infected or uninfected human T-cell lines were also investigated.

**Results:**

EOS was highly expressed at the protein level in most HTLV-1 infected T-cell lines, and was augmented after the HTLV-1 regulatory factor Tax was induced in a Tax-inducible JPX-9 cell line. Immunoprecipitation experiments demonstrated a physical interaction between EOS and the viral regulatory protein Tax, but not HBZ. Meanwhile, there was a significant decrease in EOS mRNA levels in PBMCs of HTLV-1 infected individuals irrespective of their clinical statuses. We found an inverse correlation between EOS mRNA levels and HTLV-1 PVL in ATL patients, and positive correlations between both EOS mRNA load and PVL, and EOS and HBZ mRNA load in HAM/TSP patients, whereas this correlation was not observed in other clinical statuses.

**Conclusions:**

These findings suggest that both Tax and HBZ can alter the expression of EOS through undetermined mechanisms, and dysregulated expression of EOS in PBMCs of HTLV-1 infected individuals may contribute to the pathological progression of HTLV-1-associated diseases, such as ATL and HAM/TSP.

## Background

Human T-cell leukemia virus type 1 (HTLV-1) was the first human retrovirus identified as a causative agent of human diseases, including adult T-cell leukemia/lymphoma (ATL) [1–3] and HTLV-1-associated myelopathy/tropical spastic paraparesis (HAM/TSP) [4, 5]. HAM/TSP is a chronic progressive inflammatory myelopathy, pathologically characterized by perivascular cuffing of mononuclear cells accompanied by parenchymal lymphocytic infiltration by HTLV-1 infected cells [6, 7]; it is therefore widely assumed that the immune response causes the inflammatory spinal-cord damage in HAM/TSP [8]. It is well known that elevated production of interferon-gamma (IFN-γ) is an important immunological marker of HAM/TSP pathogenesis. Indeed, in HTLV-1 infected effector memory cells of HAM/TSP patients, intracellular expression of the viral transcriptional regulator Tax is associated with rapid upregulation of IFN-γ [9]. HTLV-1-specific CD8+ cytotoxic T lymphocytes (CTLs) have the potential to secrete high levels of IFN-γ [9, 10] and are abnormally abundant in cerebrospinal fluid and spinal cord lesions [11, 12]. HTLV-1 primarily infects CD4+ T helper (Th) cells, which play a key role in adaptive immune responses [13]. More precisely, the predominant viral reservoir of HTLV-1 in infected individuals is CD4 + CD25 + CCR4+ T cells [14], which consist primarily of suppressive T cell subsets such as regulatory T cells (Tregs) and Th2 cells [15]. Importantly, two viral transcriptional regulators, Tax and HTLV-1 bZIP factor (HBZ), regulate the expression of the Foxp3 gene at the mRNA level [14, 16]; thus, the majority of HTLV-1 reservoir CD4 + CD25 + CCR4+ T cells consist of Foxp3+ Tregs [17]. Furthermore, Tax induces transcriptional changes in HTLV-1 infected T cells through loss of expression of the transcription factor Foxp3 resulting in conversion of immunosuppressive Foxp3 + CD4 + CD25 + CCR4+ Tregs to IFN-γ-producing proinflammatory Foxp3-CD4 + CD25 + CCR4+ T cells [15]. Furthermore, during HTLV-1 infection, the frequency of HTLV-1–negative Foxp3 + CD4+ T cells is positively correlated with the HTLV-1 proviral load (PVL) [18, 19], the most important risk factor for developing HAM/TSP, and CTL activity is negatively correlated with the frequency of HTLV-1–negative Foxp3 + CD4+ cells [19]. These findings suggested that T cell plasticity induced by HTLV-1 infection contributes to the pathogenesis of HAM/TSP.

EOS, which is encoded by the Ikzf4 gene, is a transcription factor that belongs to the Ikaros family and is primarily expressed in Tregs [20]. It plays a critical role in maintaining the stability and suppressive functions of Tregs [21]. Importantly, EOS forms a complex with Foxp3 that helps maintain the suppressive Treg cell phenotype, and downregulation of EOS in response to specific inflammatory signals induces a regulated transformation of Tregs into T helper-like cells which are capable of secreting proinflammatory cytokines [20]. These findings suggested that dysregulation of EOS in HTLV-1 infected Tregs is involved in the inflammatory and neurodegenerative processes of HAM/TSP. We therefore investigated whether there was an alteration in the expression of EOS in peripheral blood mononuclear cells (PBMCs) derived from HTLV-1-infected individuals with various clinical statuses, i.e. ATL, HAM/TSP, or asymptomatic carriers (ACs).

The results showed that there was a significant decrease in EOS mRNA levels in PBMCs of HTLV-1 infected individuals, irrespective of their clinical statuses. Furthermore, we found an inverse correlation between EOS mRNA levels and PVL in ATL, and positive correlations between both EOS mRNA load and PVL, and EOS and HBZ mRNA load in HAM/TSP, suggesting a possible role for EOS in the pathogenesis of HTLV-1-associated diseases.

## Methods

### Study population and preparation of clinical samples

We investigated the expression of the HTLV-1 *tax* genotype, PVL, and mRNA expression of tax, HBZ and EOS in HTLV-1 infected individuals from Okinawa, which is located in the subtropical southern-most point and comprised of remote islands off the mainland of Japan, and is highly endemic for HTLV-1 [22]. Collection of PBMCs and subsequent analyses were conducted by multiple collaborating laboratories at the Kawasaki Medical School and the University of the Ryukyus. Clinical samples from 35 patients with ATL (acute-type, *n* = 21; lymphoma-type, *n* = 6; smoldering-type, *n* = 5; chronic-type, *n* = 3), 17 patients with HAM/TSP, 26 ACs, and 14 normal uninfected controls (NCs) were analyzed. These samples were chosen at random. The clinical profiles of the HTLV-1-infected individuals and NCs that participated in this study are shown in Additional file 1: Table S1. Diagnosis of HAM/TSP and ATL was based on the World Health Organization diagnostic criteria [23] and Shimoyama criteria [24], respectively. Fresh PBMCs were isolated using Histopaque-1077 (Sigma, St. Louis, MO, USA) density gradient centrifugation, washed twice in RPMI medium, and stored in liquid nitrogen as stock lymphocytes for future use.

### Cells

In this study, we used ten HTLV-1-infected human T-cell lines (MT-1 [25], MT-2 [26], MT-4 [27], HUT102 [28], ATL43Tb [29], ATL55T [30], ED [31], TL-Om1 [32], SLB-1 [33], and ILT-M1 [34]) and three HTLV-1-uninfected T-cell lines (CEM [35], Molt-4 [35], and Jurkat [36]). These HTLV-1-infected and -uninfected T-cell lines were kindly provided by the collaborators (see Acknowledgements section) and Riken BRC (BioResource Research Center) Cell Bank (Tsukuba, Japan), respectively. To avoid passage-dependent effects and ensure valid and reproducible experimental results, all of these cell lines were properly treated by following a strict seed-stock cell-banking method proposed by American Type Culture Collection (ATCC) (https://www.atcc.org/~/media/PDFs/Marketing%20Material/Cell%20Biology/Maintaining%20High%20Standards%20in%20Cell%20Culture.ashx). MT-2 and MT-4 are chronically HTLV-1-infected cell lines derived from cord blood mononuclear cells, which were exposed to HTLV-1 isolated from patients with ATL, i.e., HTLV-1-transformed T-cell lines. MT-1, HUT102, ATL43Tb, ED, TL-Om1, ATL55T, and SLB-1 are HTLV-1-infected cell lines derived from patients with ATL. Among these ATL cell lines, only ATL55T is IL-2-dependent. ILT-M1 is an IL-2-dependent HTLV-1-infected T-cell line derived from patients with HAM/TSP. We also used the JPX-9 cell line, a Jurkat subclone generated by the stable introduction of a functional Tax expression-plasmid vector. After adding CdCl_2_ to the culture medium (final concentration: 10 μM), JPX-9 cells express biologically active Tax protein under the control of the metallothionein promoter [37]. These cells were cultured in RPMI 1640 medium, supplemented with 10% heat-inactivated fetal calf serum (FCS), 50 U/mL penicillin, and 50 μg/m streptomycin (Wako Pure Chemical, Osaka, Japan) at 37 °C under 5% CO_2_. For IL-2-dependent cell lines, 10 U/mL (for ATL55T) or 30 U/mL (for ILT-M1) of recombinant human IL-2 (Wako) was added to the culture.

### Western blotting

Whole-cell lysates were extracted from human T cell lines using Pierce RIPA Buffer (Thermo Fisher Scientific, Yokohama, Japan) with protease inhibitor cocktail (Thermo Fisher Scientific). Briefly, 1 × 10^7^ cells were washed three times with PBS, resuspended in Pierce RIPA Buffer with protease inhibitor cocktail, and then sonicated on ice using a Bioruptor® sonicator (Diagenode, Liège, Belgium), according to the manufacturer’s protocol. After centrifugation (14,000×g, 4 °C, 15 min), supernatants were collected and subjected to SDS-polyacrylamide gel electrophoresis (SDS-PAGE) followed by transfer to polyvinylidene difluoride (PVDF) membranes (pore size 0.45 μm, Merck Millipore, MA, USA) for western blotting. PVDF membranes were blocked with 5% skim milk in Tris-buffered saline containing 0.1% Tween 20 (TBS-T), and probed with anti-Tax mouse monoclonal (Lt-4) [38], anti-HBZ rat monoclonal (in-house antibody clone 4B12), anti-EOS (anti-ZNFN1A1) rabbit polyclonal (ab220329, Abcam, Tokyo, Japan), anti-β-actin rabbit polyclonal (PM053, Medical & Biological Laboratories, Nagoya, Japan), or anti-α-Tubulin rabbit polyclonal (PM054, Medical & Biological Laboratories) antibodies. PVDF membranes were washed with TBS-T and incubated with IRDye 680RD Goat anti-Mouse IgG or IRDye 800CW goat anti-rabbit IgG (LI-COR Biosciences, Lincoln, NE). After washing with TBS-T, protein levels were assayed using the Odyssey CLx Infrared Imaging System (LI-COR Biosciences).

### Plasmid construction

All primers used for plasmid construction are listed in Additional file 1: Table S2. To construct pCAGGS-P7-EOS-FLAG, the full-length EOS gene was amplified by PCR using the cDNA library from ATL55T cells as a template. Two DNA fragments of the EOS gene were separately amplified using the following primer sets, EOS fragment 1: EOS fragment 1-FOR and EOS fragment 1-REV, EOS fragment 2: EOS fragment 2-FOR and EOS fragment 2-REV, respectively. To add a tandem FLAG-tag sequence to the C-terminus of the EOS protein, DNA fragments were amplified via 2nd-PCR using EOS fragment 2-FOR and FLAG-Tandem-REV as primers and the 1st-PCR products of EOS fragment 2 as templates. Next, to fuse the full-length of the tandem FLAG-tag sequence, DNA fragments were amplified via 3rd-PCR using EOS fragment 2-FOR and FLAG-Tandem-2-REV as primers and the 2nd-PCR products of EOS fragment 2 as templates. The 3rd-PCR products of EOS fragment 2 were phosphorylated with T4 polynucleotide kinase. The EOS fragment 1 products were digested with XhoI and HindIII, and the 3rd-PCR products of EOS fragment 2 were digested with HindIII, and cloned into XhoI- and EcoRV-digested pCAGGS-P7. To construct pCAGGA-P7-HBZ-A, DNA fragments corresponding to the HBZ-A coding sequence were amplified by PCR using HBZ-FOR and HBZ-REV as primers and pTRE3G-HBZ-A [39] as the template. The PCR products were phosphorylated with T4 polynucleotide kinase, and were digested with SalI then cloned into XhoI- and EcoRV-digested pCAGGS-P7.

### Immunoprecipitation

Jurkat cells (1.5 × 10^6^ cells) were transfected with each indicated plasmid (i.e., pCAGGS-P7-EOS-FLAG, pCG-Tax-A [40], pCAGGS-P7-HBZ-A, and pEF-321 T) by electroporation and then cultured for 24 h at 37 °C. Plasmid pEF-321 T, which encodes the SV40 T antigen, was used for amplification of transfected pCAGGS-P7 and pCG. The harvested cells were solubilized using a lysis buffer (20 mM Tris-HCl [pH 7.9], 100 mM NaCl, 0.1% Triton X-100). After sonication, homogenates were centrifuged at 14,000×*g* at 4 °C for 5 min, and the supernatant fraction was used for immunoprecipitation and elution of a Flag-tagged-EOS protein. Namely, the cell extracts were incubated with ANTI-FLAG M2 Affinity Gel (A2220, SIGMA-ALDRICH Japan, Tokyo, Japan) at 4 °C for 12 h, then the resins were collected via brief centrifugation and washed twice with the lysis buffer. The resin-bound proteins were eluted by boiling in SDS-PAGE Sample Loading Buffer (GA741, TaKaRa, Shiga, Japan) and subjected to 4–15% SDS-PAGE (#4568086, Bio-Rad, Hercules, CA), followed by western blotting using anti-DDDDK-tag (anti-FLAG) rabbit polyclonal (PM020, Medical & Biological Laboratories), anti-Tax mouse monoclonal (Lt-4), and anti-HBZ rat monoclonal (4B12) antibodies. An aliquot of the cell lysates was removed before immunoprecipitation and characterized as an input (Input).

### Genomic DNA and RNA extraction and cDNA synthesis

Genomic DNA was extracted from PBMCs using the QIAamp Blood Kit (Qiagen, Tokyo, Japan). RNA was extracted from PBMCs using the RNeasy Mini Kit with on-column DNase digestion (Qiagen). cDNA was synthesized using the PrimeScript® RT Reagent Kit (TaKaRa). All reactions were performed as per the manufacturer’s instructions.

### Quantification of HTLV-1 proviral load

To examine the HTLV-1 PVL, quantitative PCR (qPCR) using primers and probes for the most conserved HTLV-1 *tax* region (amplicon length: 223 bp) was performed using 100 ng of genomic DNA (roughly equivalent to 10^4^ cells) extracted from PBMCs as previously reported [41]. The HTLV-1 PVL represents the population of infected PBMCs cells, because HTLV-1-infected cells harbor one copy of the integrated HTLV-1 provirus per cell in vivo [42]. In this method, the 5′ nuclease activity of Taq polymerase cleaves a non-extendible hybridization probe during the extension phase of PCR. This cleavage generates a specific fluorescent signal which is measured at each cycle. Based on the standard curve created by four known concentrations of template, the concentration of unknown samples can be determined. The amount of HTLV-1 proviral DNA was determined using the following formula: copy number of HTLV-1 *tax* per 1 × 10^4^ PBMCs = [(copy number of Tax)/(copy number of β − actin/2)] × 10^4^. All samples were examined in triplicate.

### Real-time quantitative reverse transcription PCR analysis

To estimate tax and HBZ mRNA expression levels, a real-time quantitative reverse transcription PCR (real-time qRT-PCR) was performed as previously described [43]. Human Ikzf4 (EOS) (Hs00223842_m1; Applied Biosystems, Foster City, CA) gene specific primers were used for Ikzf4 (EOS) quantification. The expression levels of these genes were normalized to the expression levels of human hypoxanthine phosphoribosyltransferase 1 (HPRT1) (Human HPRT1 Endogenous Control 4,333,768; Applied Biosystems). All assays were performed in triplicate.

### Statistical analysis

To test for significant differences among four different groups of subjects (HAM/TSP, ATL, ACs, and NCs), the data were statistically analyzed using one-way analysis of variance (ANOVA). Inter-group comparisons were done by Scheffe’s post hoc multiple comparisons test. The Mann-Whitney U test was used to compare the data between two groups. Correlations between variables were examined using Spearman rank correlation analyses. The results shown represent the mean ± SD where applicable. Results were considered statistically significant with *p* values < 0.05.

## Results

### Increased expression of EOS in HTLV-1-infected T-cell lines

**Fig. 1:**
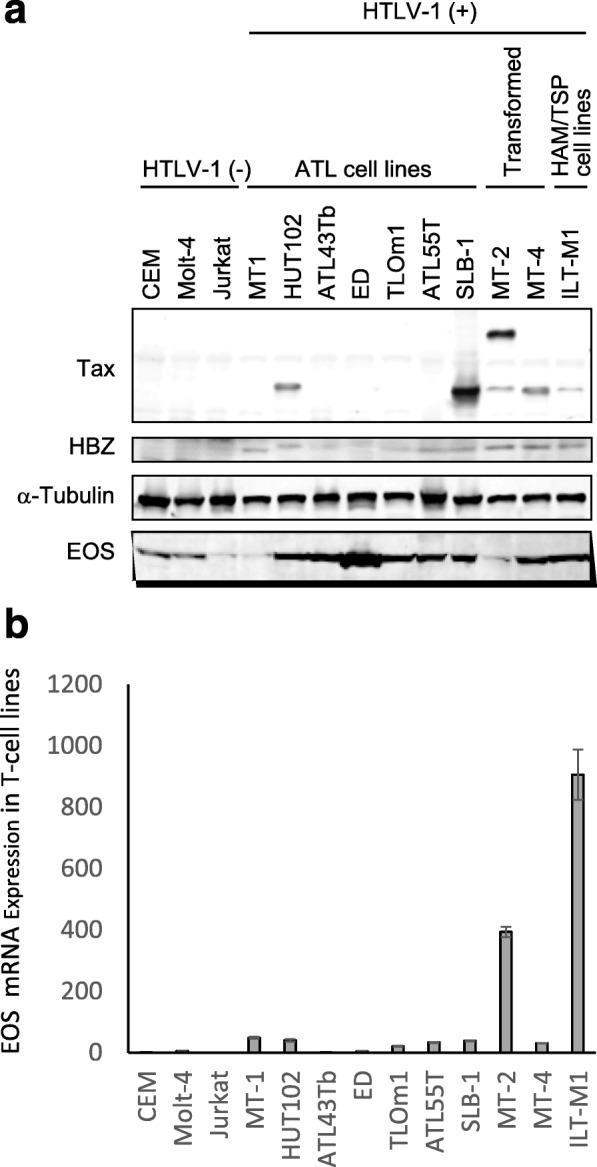
Increased expression of EOS in human T-cell leukemia virus type-1 (HTLV-1)-infected T-cell lines. **A.** Expression of EOS, Tax, and HBZ was examined by western blot in HTLV-1-infected and -uninfected T-cell lines. **B.** The expression level of EOS mRNA in HTLV-1-infected and -uninfected T-cell lines

First, we evaluated EOS protein levels in HTLV-1-infected and -uninfected human T-cell lines. As shown in Fig. 1a, EOS protein was highly expressed in most of the HTLV-1-infected T-cell lines (8 out of 10) investigated. The expression level of EOS was high irrespective of origin, i.e., in patients with ATL (MT-1, HUT102, ATL43Tb, ED, TL-Om1, ATL55T, and SLB-1), HAM/TSP (ILT-M1), or HTLV-1-transformed T-cell lines (MT-2 and MT-4). Meanwhile, the expression level of EOS in HTLV1-negative human leukemic T-cell lines (CEM, Molt4, and Jurkat), and HTLV-1 positive HTLV-1-infected MT-1 (derived from patient with ATL) and MT-2 (HTLV-1-transformed) cell lines were lower than in most HTLV-1-infected human T-cell lines (Fig. 1a). Meanwhile, the EOS mRNA expression level in these T-cell lines as evaluated by qRT-PCR showed no clear relationship with protein levels of EOS, Tax, or HBZ (Fig. 1b).

### Increased expression of EOS after induction of Tax in JPX-9 cells

**Fig. 2:**
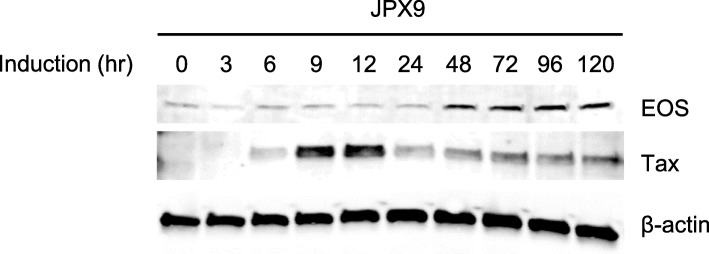
Increased expression of EOS after induction of Tax in JPX-9 cells. Expression of EOS in JPX-9 cell line was analyzed by western blotting. Tax expression was induced by adding CdCl_2_ to the culture medium (final concentration: 10 μM). EOS protein levels were increased after the induction of Tax. β-actin was used as a loading control

HTLV-1 Tax is a trans-activating protein that regulates viral gene expression, as well as many cellular genes, to enhance T-cell proliferation and cell survival. To examine whether EOS protein expression is induced by Tax, we used JPX-9 cells [37], a Jurkat subclone generated by the stable introduction of a functional Tax expression-plasmid vector, and induced Tax expression by adding CdCl_2_ to the culture medium (final concentration: 10 μM). Western blot analysis showed that treatment of JPX-9 cells with CdCl_2_ induced Tax expression, and EOS expression was augmented approximately 48 h after Tax induction, suggesting that Tax is involved in the upregulation of EOS in a human leukemic T-cell line (Fig. 2).

### EOS physically interacts with Tax but not HBZ

**Fig. 3:**
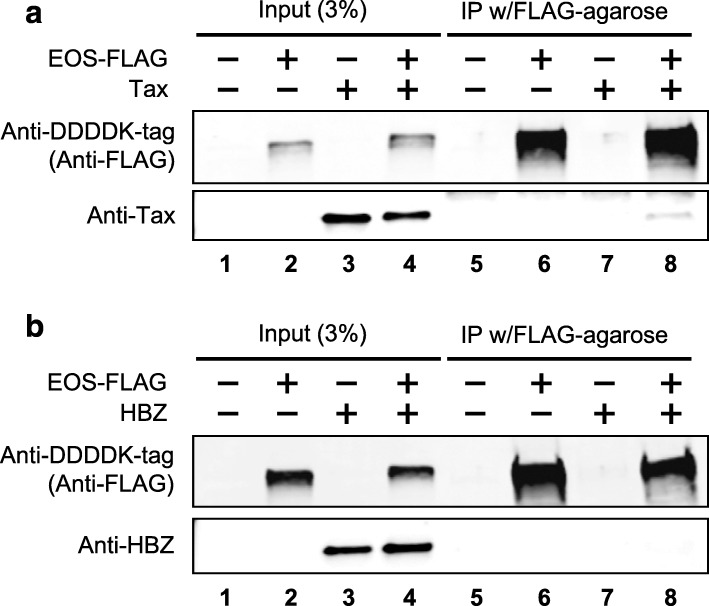
EOS physically interacts with Tax but not HBZ. Physical interactions between EOS and HTLV-1 regulatory factors (i.e., Tax or HBZ) were investigated by co-immunoprecipitation. The input samples (3%, lanes 1 to 4) and resin-bound proteins (lanes 5 to 8) were subjected to SDS-PAGE followed by western blot analysis. **A.** Western blot analysis performed on immunoprecipitated EOS protein (EOS-FLAG) revealed a weak but specific physical interaction between EOS and Tax. **B.** In contrast, no such interaction was demonstrated between EOS and HBZ.

As interactions between proteins of pathogen and host are a crucial part of the infection mechanism, we investigated physical interactions between EOS and HTLV-1 regulatory factors (i.e., Tax or HBZ) by co-immunoprecipitation. As shown in Fig. 3, we found a weak but specific physical interaction between EOS and Tax, whereas no such interaction was demonstrated between EOS and HBZ (Fig. 3).

### Significant decrease in EOS mRNA load in PBMCs of HTLV-1 infected individuals irrespective of their disease statuses

**Fig. 4:**
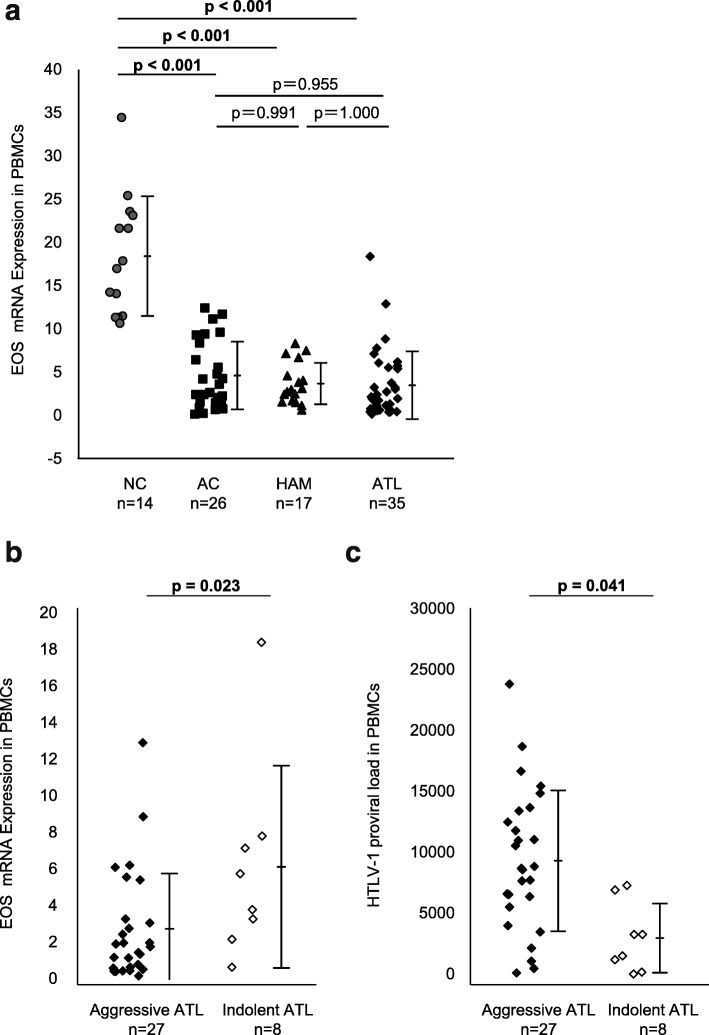
Significant decrease in EOS mRNA load in PBMCs of HTLV-1 infected individuals irrespective of their disease statuses. Expression of EOS mRNA in PBMCs was examined by real-time qRT-PCR. **A.** The expression level of EOS mRNA in HTLV-1 infected individuals was significantly lower than in NC, irrespective of their clinical statuses (i.e., AC, HAM or ATL). **B.** EOS mRNA levels in PBMCs of aggressive-ATL patients, such as acute- and lymphoma-types with poor prognosis, was significantly lower than indolent-ATL patients, such as smoldering- and chronic-types with relatively good prognosis (*p* = 0.023, Mann-Whitney U-test). **C.** HTLV-1 proviral load (PVL) in PBMCs of ATL patients. Mean HTLV-1 copy numbers per 10^4^ PBMCs were presented in each group. The HTLV-1 PVL of aggressive-ATL patients was significantly higher than that of indolent-ATL patients (*p* = 0.041, Mann-Whitney U test)

To confirm whether the in vivo expression profile of EOS mRNA was related to the clinical statuses of HTLV-1 infection, we performed qRT-PCR on the PBMCs of 35 patients with ATL (acute-type, *n* = 21; lymphoma-type, *n* = 6; smoldering-type, *n* = 5; chronic-type, *n* = 3), 17 patients with HAM/TSP, 26 ACs, and 14 NCs. The results showed that the expression level of EOS mRNA in HTLV-1 infected individuals was significantly lower than in NCs, irrespective of clinical statuses (Fig. 4a). Next, to investigate whether the in vitro expression profile of EOS mRNA indicates the clinical types of ATL associated with degree of malignancy, we compared the levels of EOS mRNA between aggressive ATL, such as acute- and lymphoma-types with poor prognosis, and indolent ATL, such as smoldering- and chronic types with relatively good prognosis. Interestingly, the EOS mRNA level in PBMCs of patients with aggressive ATL was significantly lower than in patients with indolent ATL (*p* = 0.023, Mann-Whitney U-test) (Fig. 4b). Meanwhile, the HTLV-I PVL from aggressive-ATL patients was significantly higher than in indolent ATL patients (*p* = 0.041, Mann-Whitney U test) (Fig. 4c).

### Comparison of EOS mRNA levels with tax and HBZ mRNA levels, and with PVL, among HTLV-1 infected individuals with different clinical statuses

**Fig. 5:**
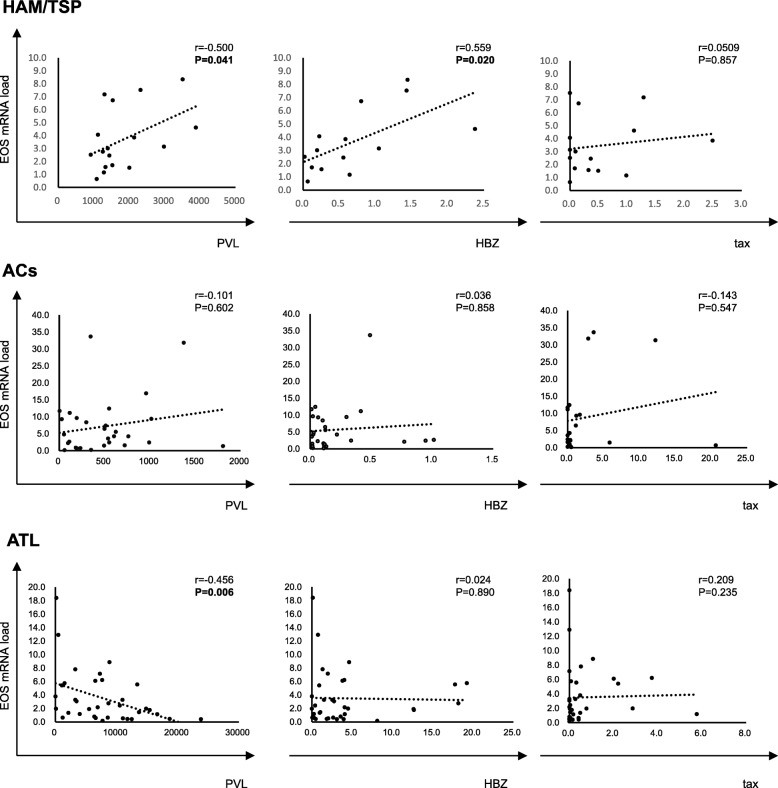
Comparison of EOS mRNA levels with tax and HBZ mRNA levels as well as with HTLV-1 PVL among HTLV-1 infected individuals with different clinical statuses. Positive correlations were observed between both EOS mRNA load and PVL, and EOS and HBZ mRNA load in HAM/TSP patients, but not in patients with other clinical statuses (first row, left and middle panels). In ACs, the EOS mRNA level in PBMCs did not correlate with HTLV-1 PVL or Tax and HBZ mRNA expression (second row). There is an inverse correlation between EOS mRNA load and PVL in ATL patients, but not in patients with other clinical statuses (third row, left panel)

To investigate whether the decreased EOS mRNA load in PBMCs of HTLV-1 infected individuals is associated with viral gene expression and the number of infected cells in vivo, we quantified the expression of HTLV-1 regulatory genes, tax and HBZ, as well as PVL in PBMCs derived from HTLV-1 infected individuals with different clinical statuses. We found an inverse correlation between EOS mRNA load and PVL in ATL patients, but not in patients with infections presenting with other clinical statuses (Fig. 5, third row, left panel). We also found positive correlations between both EOS mRNA load and PVL and EOS and HBZ mRNA load in HAM/TSP patients, but not in patients with other clinical statuses (Fig. 5, first row, left and middle panels). However, in ACs, the EOS mRNA level in PBMCs did not correlate with HTLV-1 PVL, or Tax and HBZ mRNA expression (Fig. 5, second row).

## Discussion

The members of the Ikaros family of zinc finger (Ikzf) transcription factors have a crucial role not only in immune cell development and homeostasis, but also pathological conditions in human [21, 44]. It has been reported that deregulation of IKAROS, the founding member of the family encoded by the Ikzf1 gene, results in leukemia in both mice and human [45], whereas downregulation of EOS, encoded by the Ikzf4 gene, induces a regulated conversion of Tregs into T helper-like cells that are capable of secreting proinflammatory cytokines in response to specific inflammatory signals [20]. As HTLV-1 can cause both leukemia (i.e., ATL) and inflammatory disease (i.e., HAM/TSP), and conversion of Tregs to T helper-like cells has been reported in patients with HAM/TSP [15], we investigated in this study whether the expression level of EOS is associated with HTLV-1 infection and progression to disease.

Our data indicated that most HTLV-1 infected T-cell lines (i.e., 8 out of 10 tested) exhibited high expression of the EOS protein. In HTLV-1 infection, it is well established that both Tax and HBZ play a key role in the transactivation of viral and cellular genes for HTLV-1 replication and pathogenesis [46]. To date, several genes including transcription factors and cell signaling mediators have been reported to be Tax targets [39, 47, 48]. Consistent with these findings, our data showed that the expression of EOS protein was augmented after Tax induction in JPX-9 cells, and Tax, but not HBZ, physically interacts with the EOS protein. Tax-mediated expression of EOS was further supported by our previous microarray study, which indicated that the EOS gene was induced after induction of Tax but not HBZ in Jurkat T-cells [39]. These findings suggest that EOS is one of the cellular targets of Tax. Interestingly, however, among those HTLV-1 infected T-cell lines which exhibited high expression of the EOS protein, gene expression (mRNA) levels of EOS were relatively low except for two T-cell lines (MT-2 and ILT-M1), thus did not correlate well with protein levels. These results suggest that stable expression of EOS is not only dependent on Tax-mediated regulation, but also on various cellular processes including protein degradation and/or epigenetic mechanisms, which might be altered in different HTLV-1 infected T-cell lines.

In PBMCs of HTLV-1 infected individuals, EOS mRNA levels was considered low when compared with NCs, regardless of clinical statuses, i.e., ATL, HAM/TSP, or ACs. However, since ex vivo data obtained using patient samples may provide more valuable insight into the clinical significance of EOS with respect to the risk of HTLV-1 associated diseases, we assume that this is the most fascinating and important finding of the present study. In ATL patients, there was an inverse correlation between EOS mRNA levels and HTLV-1 PVL, suggesting lower levels of EOS mRNA expression in ATL cells through an as yet undetermined mechanism. ATL is divided into four clinical subtypes, i.e., acute, lymphoma, chronic, and smoldering [24], and patients with acute, lymphoma, and chronic type (with unfavorable prognostic factors) are categorized as suffering from aggressive ATL, whereas those with the chronic type without unfavorable prognostic factors, and the smoldering type are categorized as suffering from indolent ATL [49]. Treatment of ATL is usually determined based on these clinical subtypes and prognostic factors, and the presence of an aggressive or indolent ATL is critical when making clinical decisions. As our data showed significantly lower EOS mRNA levels in patients with aggressive ATL than in patients with indolent ATL, EOS mRNA levels may serve as an additional diagnostic marker for aggressive or indolent ATL.

In contrast to ATL patients, we found a positive correlation between EOS mRNA load and PVL in HAM/TSP patients. Moreover, consistent with our previous study [43], we also found a positive correlation between HBZ mRNA load and PVL. As a consequence, the expression levels of EOS mRNA showed a significant positive correlation with HBZ, but not with Tax, expression. Since we previously reported that the expression levels of HBZ but not Tax mRNA positively correlated with disease severity in HAM/TSP patients [43], it is conceivable that HBZ, which has a bimodal function at the mRNA and protein levels, increases the level of EOS mRNA in PBMCs of HAM/TSP patients. However, there was conversely a significant decrease in EOS mRNA level in PBMCs of HTLV-1 infected individuals irrespective of their clinical statuses, i.e., ATL, HAM/TSP, or ACs. Since the EOS gene is regulated by various cellular processes, including protein degradation and/or epigenetic mechanisms, one possible explanation for the decreased levels of EOS mRNA in PBMCs is the differing methylation status of the EOS regulatory regions between HTLV-1 infected individuals and NCs, especially the Treg-specific methylation region (TSDR). Namely, if the demethylation rate is lower in PBMCs from HTLV-1 infected individuals than in NCs, decreased demethylation may be associated with decreased expression. Indeed, Anderson et al. have already reported that the demethylation rate of the Foxp3 TSDR is lower in PBMCs and CD4 + CD25+ T cells from HAM/TSP patients than in uninfected normal controls, and decreased TSDR demethylation is associated with decreased functional suppression via Tregs in HAM/TSP patients [50].

It is well established that HTLV-1 infection induces an abnormal frequency and phenotype of Foxp3 + CD4+ T cells in infected individuals [51]. In particular, HTLV-1–negative Foxp3 + CD4+ Tregs are negatively correlated with CTL activity [19], and positively correlated with HTLV-1 PVL [18, 19]. These findings suggest that HTLV-1–negative Foxp3 + CD4+ Tregs are the primary determinant of the efficiency of T cell–mediated immune control of HTLV-1. Both Tax and HBZ are associated with this process. Namely, HBZ RNA promotes T cell proliferation [52] and induces Foxp3 expression [16], which results in the increased frequency of HTLV-1–negative Foxp3 + CD4+ Tregs, whereas Tax induces the conversion of immunosuppressive Foxp3 + CD4 + CD25 + CCR4+ Tregs to IFN-γ-producing proinflammatory Foxp3-CD4 + CD25 + CCR4+ T cells via transcriptional changes in HTLV-1 infected T cells through loss of Foxp3 [15]. Importantly, in addition to neurological symptoms, some HAM/TSP cases also exhibit autoimmune-like disorders, such as uveitis, arthritis, T-lymphocyte alveolitis, polymyositis, and Sjögren syndrome [53], and both Tax and HBZ are associated with the inflammatory process of HAM/TSP via regulation of Treg and T helper cells [54]. Because increased HBZ levels increased the risk of developing HAM/TSP [55], it may be possible that HBZ induces Foxp3, which reduces CTL activity, which, in turn, increases the HTLV-1 PVL.

Meanwhile, downregulation of EOS impaired and reduced Foxp3-mediated gene repression, thereby leading to Treg dysfunction and autoimmunity. EOS-deficient (EOS−/−) mice developed more severe experimental autoimmune encephalomyelitis, which is the most commonly used experimental model for the human inflammatory demyelinating disease called multiple sclerosis, which is pathologically similar to HAM/TSP and presents with an increased abundance of effector T cells in the periphery and central nervous system [56], Furthermore, EOS reduces Foxp3 acetylation and enhances K48-linked polyubiquitylation [57], EOS interacts with Foxp3 and mediates its gene silencing activity [20], and selective deletion of EOS in Tregs leads to loss of suppressive function and development of systemic autoimmunity [58]. Although further studies are required to determine the in vivo significance, these findings suggest the possibility that HTLV-1 contribute to development of inflammatory conditions observed in HAM/TSP, by altering Treg status via modulating both Foxp3 levels and biological activities of EOS.

## Conclusion

In conclusion, our study identified dysregulated expression of EOS in HTLV-1-infected individuals. Although further studies are needed to define the in vivo significance of EOS expression and its participation in disease processes during HTLV-1 infection, dysregulated expression of EOS may be the key to understanding the pathogenesis of HTLV-1 associated diseases.

## Supplementary information

**Additional file 1: Table S1.** Characteristics of HTLV-1-infected and -uninfected study participants. **Table S2.** Primer sequences for plasmid construction.

## Data Availability

All data generated or analyzed during this study are included herein.

## Abbreviations

ACs: Asymptomatic carriers
ATL: Adult T-cell leukemia
HAM/TSP: HTLV-1-associated myelopathy/tropical spastic paraparesis
HTLV-1: Human T-cell leukemia virus type-1
NCs: Normal uninfected controls

## References

[1] Poiesz BJ, Ruscetti FW, Gazdar AF, Bunn PA, Minna JD, Gallo RC (1980). Detection and isolation of type C retrovirus particles from fresh and cultured lymphocytes of a patient with cutaneous T-cell lymphoma. Proc Natl Acad Sci U S A.

[2] Hinuma Y, Nagata K, Hanaoka M, Nakai M, Matsumoto T, Kinoshita KI, Shirakawa S, Miyoshi I (1981). Adult T-cell leukemia: antigen in an ATL cell line and detection of antibodies to the antigen in human sera. Proc Natl Acad Sci U S A.

[3] Yoshida M, Seiki M, Yamaguchi K, Takatsuki K (1984). Monoclonal integration of human T-cell leukemia provirus in all primary tumors of adult T-cell leukemia suggests causative role of human T-cell leukemia virus in the disease. Proc Natl Acad Sci U S A.

[4] Gessain A, Barin F, Vernant JC, Gout O, Maurs L, Calender A, de The G (1985). Antibodies to human T-lymphotropic virus type-I in patients with tropical spastic paraparesis. Lancet.

[5] Osame M, Usuku K, Izumo S, Ijichi N, Amitani H, Igata A, Matsumoto M, Tara M (1986). HTLV-I associated myelopathy, a new clinical entity. Lancet.

[6] Matsuoka E, Takenouchi N, Hashimoto K, Kashio N, Moritoyo T, Higuchi I, Isashiki Y, Sato E, Osame M, Izumo S (1998). Perivascular T cells are infected with HTLV-I in the spinal cord lesions with HTLV-I-associated myelopathy/tropical spastic paraparesis: double staining of immunohistochemistry and polymerase chain reaction in situ hybridization. Acta Neuropathol.

[7] Izumo S, Umehara F, Osame M (2000). HTLV-I-associated myelopathy. Neuropathology.

[8] Osame M (2002). Pathological mechanisms of human T-cell lymphotropic virus type I-associated myelopathy (HAM/TSP). J Neurovirol.

[9] Hanon E, Goon P, Taylor GP, Hasegawa H, Tanaka Y, Weber JN, Bangham CR (2001). High production of interferon gamma but not interleukin-2 by human T-lymphotropic virus type I-infected peripheral blood mononuclear cells. Blood.

[10] Kubota R, Kawanishi T, Matsubara H, Manns A, Jacobson S (2000). HTLV-I specific IFN-gamma+ CD8+ lymphocytes correlate with the proviral load in peripheral blood of infected individuals. J Neuroimmunol.

[11] Nagai M, Yamano Y, Brennan MB, Mora CA, Jacobson S (2001). Increased HTLV-I proviral load and preferential expansion of HTLV-I tax-specific CD8+ T cells in cerebrospinal fluid from patients with HAM/TSP. Ann Neurol.

[12] Matsuura E, Yamano Y, Jacobson S (2010). Neuroimmunity of HTLV-I infection. J NeuroImmune Pharmacol.

[13] Matsuoka M, Jeang KT (2007). Human T-cell leukaemia virus type 1 (HTLV-1) infectivity and cellular transformation. Nat Rev Cancer.

[14] Yamano Y, Takenouchi N, Li HC, Tomaru U, Yao K, Grant CW, Maric DA, Jacobson S (2005). Virus-induced dysfunction of CD4+CD25+ T cells in patients with HTLV-I-associated neuroimmunological disease. J Clin Invest.

[15] Araya N, Sato T, Ando H, Tomaru U, Yoshida M, Coler-Reilly A, Yagishita N, Yamauchi J, Hasegawa A, Kannagi M (2014). HTLV-1 induces a Th1-like state in CD4+CCR4+ T cells. J Clin Invest.

[16] Satou Y, Yasunaga J, Zhao T, Yoshida M, Miyazato P, Takai K, Shimizu K, Ohshima K, Green PL, Ohkura N (2011). HTLV-1 bZIP factor induces T-cell lymphoma and systemic inflammation in vivo. PLoS Pathog.

[17] Sakaguchi S, Ono M, Setoguchi R, Yagi H, Hori S, Fehervari Z, Shimizu J, Takahashi T, Nomura T (2006). Foxp3+ CD25+ CD4+ natural regulatory T cells in dominant self-tolerance and autoimmune disease. Immunol Rev.

[18] Hayashi D, Kubota R, Takenouchi N, Nakamura T, Umehara F, Arimura K, Izumo S, Osame M (2008). Accumulation of human T-lymphotropic virus type I (HTLV-I)-infected cells in the cerebrospinal fluid during the exacerbation of HTLV-I-associated myelopathy. J Neurovirol.

[19] Toulza F, Heaps A, Tanaka Y, Taylor GP, Bangham CR (2008). High frequency of CD4+FoxP3+ cells in HTLV-1 infection: inverse correlation with HTLV-1-specific CTL response. Blood.

[20] Pan F, Yu H, Dang EV, Barbi J, Pan X, Grosso JF, Jinasena D, Sharma SM, EM MC, Getnet D (2009). Eos mediates Foxp3-dependent gene silencing in CD4+ regulatory T cells. Science.

[21] Heizmann B, Kastner P, Chan S (2018). The Ikaros family in lymphocyte development. Curr Opin Immunol.

[22] Sakihama Shugo, Saito Mineki, Kuba-Miyara Megumi, Tomoyose Takeaki, Taira Naoya, Miyagi Takashi, Hayashi Masaki, Kinjo Shigeko, Nakachi Sawako, Tedokon Iori, Nishi Yukiko, Tamaki Keita, Morichika Kazuho, Uchihara Jun-nosuke, Morishima Satoko, Karube Ken-nosuke, Tanaka Yuetsu, Masuzaki Hiroaki, Fukushima Takuya (2017). Human T-cell leukemia virus type I Tax genotype analysis in Okinawa, the southernmost and remotest islands of Japan: Different distributions compared with mainland Japan and the potential value for the prognosis of aggressive adult T-cell leukemia/lymphoma. Leukemia Research.

[23] Osame M (1990). Review of WHO Kagoshima meeting and diagnostic guidelines for HAM/TSP.

[24] Shimoyama M (1991). Diagnostic criteria and classification of clinical subtypes of adult T-cell leukaemia-lymphoma. A report from the lymphoma study group (1984-87). Br J Haematol.

[25] Miyoshi I, Kubonishi I, Sumida M, Hiraki S, Tsubota T, Kimura I, Miyamoto K, Sato J (1980). A novel T-cell line derived from adult T-cell leukemia. Gan.

[26] Miyoshi I, Kubonishi I, Yoshimoto S, Akagi T, Ohtsuki Y, Shiraishi Y, Nagata K, Hinuma Y (1981). Type C virus particles in a cord T-cell line derived by co-cultivating normal human cord leukocytes and human leukaemic T cells. Nature.

[27] Harada S, Koyanagi Y, Yamamoto N (1985). Infection of human T-lymphotropic virus type-I (HTLV-I)-bearing MT-4 cells with HTLV-III (AIDS virus): chronological studies of early events. Virology.

[28] Gazdar AF, Carney DN, Bunn PA, Russell EK, Jaffe ES, Schechter GP, Guccion JG (1980). Mitogen requirements for the in vitro propagation of cutaneous T-cell lymphomas. Blood.

[29] Hattori T, Asou N, Suzushima H, Takatsuki K, Tanaka K, Naito K, Natori H, Oizumi K (1991). Leukaemia of novel gastrointestinal T-lymphocyte population infected with HTLV-I. Lancet.

[30] Maeda M, Arima N, Daitoku Y, Kashihara M, Okamoto H, Uchiyama T, Shirono K, Matsuoka M, Hattori T, Takatsuki K (1987). Evidence for the interleukin-2 dependent expansion of leukemic cells in adult T cell leukemia. Blood.

[31] Maeda M, Shimizu A, Ikuta K, Okamoto H, Kashihara M, Uchiyama T, Honjo T, Yodoi J (1985). Origin of human T-lymphotrophic virus I-positive T cell lines in adult T cell leukemia. Analysis of T cell receptor gene rearrangement. J Exp Med.

[32] Sugamura K, Fujii M, Kannagi M, Sakitani M, Takeuchi M, Hinuma Y (1984). Cell surface phenotypes and expression of viral antigens of various human cell lines carrying human T-cell leukemia virus. Int J Cancer.

[33] Koeffler HP, Chen IS, Golde DW (1984). Characterization of a novel HTLV-infected cell line. Blood.

[34] Tanaka Y, Takahashi Y, Tanaka R, Kodama A, Fujii H, Hasegawa A, Kannagi M, Ansari AA, Saito M (2014). Elimination of human T cell leukemia virus type-1-infected cells by neutralizing and antibody-dependent cellular cytotoxicity-inducing antibodies against human t cell leukemia virus type-1 envelope gp46. AIDS Res Hum Retroviruses.

[35] Drexler HG, Gaedicke G, Minowada J (1987). T-leukemia cell lines CCRF-CEM, HPB-ALL, JM and MOLT-4: changes in isoenzyme profiles during induction of differentiation. Blut.

[36] Gillis S, Watson J (1980). Biochemical and biological characterization of lymphocyte regulatory molecules. V. Identification of an interleukin 2-producing human leukemia T cell line. J Exp Med.

[37] Nagata K, Ohtani K, Nakamura M, Sugamura K (1989). Activation of endogenous c-fos proto-oncogene expression by human T-cell leukemia virus type I-encoded p40tax protein in the human T-cell line, Jurkat. J Virol.

[38] Lee B, Tanaka Y, Tozawa H (1989). Monoclonal antibody defining tax protein of human T-cell leukemia virus type-I. Tohoku J Exp Med.

[39] Naito T, Yasunaga JI, Mitobe Y, Shirai K, Sejima H, Ushirogawa H, Tanaka Y, Nakamura T, Hanada K, Fujii M (2018). Distinct gene expression signatures induced by viral transactivators of different HTLV-1 subgroups that confer a different risk of HAM/TSP. Retrovirology.

[40] Yasuma K, Yasunaga J, Takemoto K, Sugata K, Mitobe Y, Takenouchi N, Nakagawa M, Suzuki Y, Matsuoka M (2016). HTLV-1 bZIP factor impairs anti-viral immunity by inducing co-inhibitory molecule, T cell immunoglobulin and ITIM domain (TIGIT). PLoS Pathog.

[41] Nagai M, Usuku K, Matsumoto W, Kodama D, Takenouchi N, Moritoyo T, Hashiguchi S, Ichinose M, Bangham CR, Izumo S, Osame M (1998). Analysis of HTLV-I proviral load in 202 HAM/TSP patients and 243 asymptomatic HTLV-I carriers: high proviral load strongly predisposes to HAM/TSP. J Neurovirol.

[42] Cook LB, Rowan AG, Melamed A, Taylor GP, Bangham CR (2012). HTLV-1-infected T cells contain a single integrated provirus in natural infection. Blood.

[43] Saito M, Matsuzaki T, Satou Y, Yasunaga J, Saito K, Arimura K, Matsuoka M, Ohara Y (2009). In vivo expression of the HBZ gene of HTLV-1 correlates with proviral load, inflammatory markers and disease severity in HTLV-1 associated myelopathy/tropical spastic paraparesis (HAM/TSP). Retrovirology.

[44] Powell MD, Read KA, Sreekumar BK, Oestreich KJ (2019). Ikaros zinc finger transcription factors: regulators of cytokine signaling pathways and CD4(+) T helper cell differentiation. Front Immunol.

[45] Olsson L, Johansson B (2015). Ikaros and leukaemia. Br J Haematol.

[46] Matsuoka M, Yasunaga J (2013). Human T-cell leukemia virus type 1: replication, proliferation and propagation by tax and HTLV-1 bZIP factor. Curr Opin Virol.

[47] Sun SC, Yamaoka S (2005). Activation of NF-kappaB by HTLV-I and implications for cell transformation. Oncogene.

[48] Boxus M, Twizere JC, Legros S, Dewulf JF, Kettmann R, Willems L (2008). The HTLV-1 tax interactome. Retrovirology.

[49] Watanabe T (2017). Adult T-cell leukemia: molecular basis for clonal expansion and transformation of HTLV-1-infected T cells. Blood.

[50] Anderson MR, Enose-Akahata Y, Massoud R, Ngouth N, Tanaka Y, Oh U, Jacobson S (2014). Epigenetic modification of the FoxP3 TSDR in HAM/TSP decreases the functional suppression of Tregs. J NeuroImmune Pharmacol.

[51] Satou Y, Utsunomiya A, Tanabe J, Nakagawa M, Nosaka K, Matsuoka M (2012). HTLV-1 modulates the frequency and phenotype of FoxP3+CD4+ T cells in virus-infected individuals. Retrovirology.

[52] Satou Y, Yasunaga J, Yoshida M, Matsuoka M (2006). HTLV-I basic leucine zipper factor gene mRNA supports proliferation of adult T cell leukemia cells. Proc Natl Acad Sci U S A.

[53] Nakagawa M, Izumo S, Ijichi S, Kubota H, Arimura K, Kawabata M, Osame M (1995). HTLV-I-associated myelopathy: analysis of 213 patients based on clinical features and laboratory findings. J Neurovirol.

[54] Miyazato P, Matsuoka M (2014). Human T-cell leukemia virus type 1 and Foxp3 expression: viral strategy in vivo. Int Immunol.

[55] Saito M (2014). Neuroimmunological aspects of human T cell leukemia virus type 1-associated myelopathy/tropical spastic paraparesis. J Neurovirol.

[56] Rieder SA, Metidji A, Glass DD, Thornton AM, Ikeda T, Morgan BA, Shevach EM (2015). Eos is redundant for regulatory T cell function but plays an important role in IL-2 and Th17 production by CD4+ conventional T cells. J Immunol.

[57] Bettini ML, Pan F, Bettini M, Finkelstein D, Rehg JE, Floess S, Bell BD, Ziegler SF, Huehn J, Pardoll DM, Vignali DA (2012). Loss of epigenetic modification driven by the Foxp3 transcription factor leads to regulatory T cell insufficiency. Immunity.

[58] Gokhale AS, Gangaplara A, Lopez-Occasio M, Thornton AM, Shevach EM. Selective deletion of Eos (Ikzf4) in T-regulatory cells leads to loss of suppressive function and development of systemic autoimmunity. J Autoimmun. 2019;102300. 10.1016/j.jaut.2019.06.011 PMC11046398 31296356

