# Supplementary material for: EOS, an Ikaros family zinc finger transcription factor, interacts with the HTLV-1 oncoprotein Tax and is downregulated in peripheral blood mononuclear cells of HTLV-1-infected individuals, irrespective of clinical statuses
Source: Virol J. 2019 Dec 19;16:160. doi: 10.1186/s12985-019-1270-1 (PMC6923961; doi:10.1186/s12985-019-1270-1)
Supplement: Supplementary file 1 — Additional file 1: Table S1. Characteristics of HTLV-1-infected and -uninfected study participants. Table S2. Primer sequences for plasmid construction. [file 12985_2019_1270_MOESM1_ESM.docx]

**Supplemental Table 1: Characteristics of HTLV-1-infected and -uninfected study participants.**

|  | NCs  (n=14) | ACs  (n=26) | HAM/TSP  (n=17) | ATL  (n=35) |
| --- | --- | --- | --- | --- |
| Age | 52.1 ± 16. | 54.3 ± 17.4 | 62.7 ± 11.7 | 66.7 ± 13.6 |
| Sex, n (%) |  |  |  |  |
| Male | 8 (57.1) | 11 (42.3) | 6 (35.3) | 16 (45.7) |
| Female | 6 (42.9) | 15 (57.7) | 11 (64.7) | 19 (54.3) |
| ^a^ HTLV-1 proviral load  (Median) | N/A | 479.6 ± 441.6  (423.5) | 1826.8 ± 877.0  (1442.0) | 7850.8 ± 5877.8  (7286.0) |

NCs: normal uninfected controls

ACs: asymptomatic HTLV-1 carriers

HAM/TSP: HTLV-1 associated myelopathy/tropical spastic paraparesis

ATL: adult T-cell leukemia/lymphoma

The results represent the mean ± SD.

^a^ HTLV-1 Tax copy number per 10^4^ PBMCs.

N/A: not applicable

## Supplemental Table 2: Primer sequences for plasmid construction.

| Primer name | Direction | Sequences (5' to 3') |
| --- | --- | --- |
| EOS fragment 1-FOR | Forward | GCACGCGCTAG**CTCGAG**CCACCACCATGCATACACCACCCGCACTCCCT |
| EOS fragment 1-REV | Reverse | TGGCCAGCCA**AAGCTT**GGGCTTCAGT |
| EOS fragment 2-FOR | Forward | ACTGAAGCCC**AAGCTT**TGGCTGGCCA |
| EOS fragment 2-REV | Reverse | TCGTCGTCATCCTTGTAGTCTTTACCGCCCACCTTATGCTCCCCCCGGACA |
| FLAG-Tandem-REV | Reverse | GTCATCCTTGTAGTCCCCCTTGTCGTCGTCATCCTTGTAGTCTT |
| FLAG-Tandem-2-REV | Reverse | CCGAGATCTCTACTTGTCGTCGTCATCCTTGTAGTCCC |
| HBZ-FOR | Forward | ACGCACGC**GTCGAC**GCCACCACCATGGCGGCCTCAGGGCTGT |
| HBZ-REV | Reverse | TGGAAGATCTTATTGCAACCACATCGCCTC |

CTCGAG, AAGCTT, and GTCGAC sequences (bold letters) are the respective restriction sites for XhoI, HindIII, and SalI.

Underlines indicate the FLAG-tag sequences. Double underline indicate the Kozak sequences.
